# Subtypes of cytotoxic lymphocytes and natural killer cells infiltrating cancer nests correlate with prognosis in patients with vulvar squamous cell carcinoma

**DOI:** 10.1007/s00262-013-1511-x

**Published:** 2013-12-25

**Authors:** Jacek Jan Sznurkowski, Anton Żawrocki, Wojciech Biernat

**Affiliations:** 1 Department of Oncological Surgery, The Medical University of Gdańsk, ul. Smoluchowskiego 17, 80-214 Gdańsk, Poland; 2 Department of Pathology, The Medical University of Gdańsk, Gdańsk, Poland

**Keywords:** Vulvar cancer, vSCC, CD56+, Granzyme B, Prognosis, TILs

## Abstract

**Objective:**

Adaptive immune effectors do not influence prognosis in vulvar squamous cell carcinoma (vSCC). Therefore, we tried to clarify the prognostic role of innate immunity and granzyme B-dependent cytotoxicity as defined by intratumoral infiltrates of natural killer cells (CD56+) and lymphocytes expressing granzyme B (GrB+).

**Methods:**

We analyzed 76 primary vSCCs and 35 lymph node metastases that were obtained from 76 patients with a full clinical history. The distribution and density of GrB+ and CD56+ cells within cancer tissues were evaluated by immunohistochemistry and correlated with clinicopathological features, commonly recognized prognostic factors and overall survival (OS).

**Results:**

CD56+ cells were mostly detected within the cancer nests, while GrB+ cells were predominant in the tumor stroma. Intraepithelial (IE) CD56+ infiltrates at the primary site were correlated with depth of invasion (*r* = 0.339, *p* = 0.003) and recurrence (*r* = 0.295, *p* = 0.011), while IE GrB+ infiltrates were correlated with tumor grade (*r* = 0.304, *p* = 0.009) and age (*r* = 0.333, *p* = 0.004). The primary cancer nests of metastatic patients were infiltrated more by intraepithelial (IE) CD56+ cells than were those of the non-metastatic patients (*p* = 0.05). The median OS was 41.16 months (range 1.7–98.43). High IE GrB+ infiltrates predicted longer OS among patients without metastases (*p* = 0.028). High IE CD56+ infiltrates were correlated with longer OS in metastatic cases (*p* = 0.009).

**Conclusion:**

The combined cytotoxicity of innate and adaptive immune effectors infiltrating cancer nests (IE GrB+) predicts an improved clinical outcome among non-metastatic vSCC patients. The functional status of prognostic IE CD56+ infiltrates in immune escaped (metastatic) tumors requires further investigation.

## Introduction

Vulvar cancer has an incidence of 1–2 per 100,000 women per year and represents 3–5 % of all gynecological malignancies [1–3]. Squamous cell carcinoma (SCC) is the predominant malignancy at this site, accounting for approximately 85–90 % of vulvar cancers [4, 5].

Treatment of advanced stage (FIGO III and IV) vulvar cancer patients may be ineffective, even with chemotherapy and radiotherapy [6, 7]. Therefore, new approaches are required including immunotherapy and greater knowledge of the factors influencing prognosis.

Tumor infiltrating lymphocytes (TILs) are considered to be a manifestation of the host immune response against cancer cells [8]. CD8 + lymphocytes represent an important subpopulation of cytotoxic T lymphocytes and are the most likely effector TILs of adaptive anti-tumor immunity [9].

Innate anti-tumor immunity is mediated by cells or soluble factors that naturally exist in the tumor microenvironment. Among hematopoietic cells, natural killer (NK) (CD3−CD56+) and NK/T (CD3+CD56+) cells have the natural ability to eliminate tumor-cell targets [10, 11].

The cytotoxic function of all immune cells critically depends on serine proteases known as granzymes. As granzyme B is one of the most abundant granzymes, granzyme B-mediated cytotoxicity has been intensively studied [12, 13].

GrB-induced cell death has traditionally been viewed as a primary mechanism that is used by adaptive (CD8+) as well as innate (NK/NKT) effectors to eliminate harmful target cells including tumor cells [12].

Recently, the status of adaptive immunity represented by CD8+, CD4+ and FOXP3+ T cells was found to lack prognostic significance in vSCC [14–16]. Therefore, the purpose of this study was to clarify the prognostic roles of innate immune effectors represented by CD56+ cells and granzyme B-dependent cytolysis.

## Materials and methods

This retrospective study was approved by the Polish Ministry of Science and Higher Education review board. The board determined that further informed consent was not required, as all patients provided informed consent for tissue sampling prior to surgical treatment, including written consent for the storage of their information in the hospital database and the use of their information for research.

### Patients and specimens

This study included 76 patients with verified histopathological data and full clinical histories. The clinico-pathological characteristics of this group were carefully described in our two previous manuscripts (section: materials and methods) analyzing the expression of indoleamine 2,3-dioxygenase (IDO) and the infiltration of CD8+, CD4+ and FOXP3+ T lymphocytes in vSCC [14, 15]. The median age of the patients was 69.5 years (range 36–85), the median duration of follow-up was 51.23 months (range 6.33–135.5), and the median overall survival was 41.16 months (range 1.7–98.43) [14, 15].

The immunohistochemical staining and histological analyses were performed on paraffin-embedded material consisting of 76 primary tumors and additional lymph node metastases from 35 patients.

### Antibodies

A mouse anti-human monoclonal antibody against CD56 (cat. No NCL-CD56-1B; concentration 1:400) and a mouse anti-human polyclonal antibody against Granzyme B (cat. No 760-4283; ready to use) were obtained from Ventana Medical Systems, Inc.

### Immunohistochemistry

The immunohistochemical staining for CD56 and GrB was performed according to the following protocol. Serial sections with a thickness of 4 μm were cut, deparaffinized and subjected to a heat-induced epitope retrieval step before incubation with the primary antibodies. Sections were immersed in target retrieval solution (pH 6.0 for CD56, Dako Cytomation, Denmark) and heated in a pressure cooker. The slides were incubated for 90 min with the primary antibodies.

The reaction was visualized by the Novolink Polymer Detection System (Novocastra Laboratories). Appropriate positive (normal CD56 and GrB status) and negative controls (the primary antibody was replaced with normal mouse IgG at an appropriate dilution) were included for each case. The immunohistochemical results were evaluated by two independent pathologists who did not have access to the clinical data.

### Evaluation and classification of CD56+ and GrB+ cells

The degree of immune cell infiltration was determined for more than 10 high-power (×400) microscopic fields for each tissue sample. Then, five areas with the densest lymphocyte distribution (hot spots) were selected and microphotographs were taken. The quantitative analysis was performed with Multiscan 14.2 software. The numbers of CD56+ and GrB+ cells were counted exclusively within the primary tumor cancer cell nest. For each case, the mean index of CD56+ and GrB+ cells per single high-power field was counted and then statistically analyzed.

Patients were divided into CD56 low- and high-intensity groups (low CD56+ and high CD56+, respectively) and GrB low- and high-intensity groups (low GrB+ and high GrB+, respectively) based on median cell number (cut-off point) to determine differences in overall survival among these groups [12, 14, 15, 17–19].

### Statistical analysis

The statistical analysis was performed using the chi-square test or Fisher’s exact probability test, followed by the Kruskal–Wallis test. Correlations and differences between variables were assessed using the Spearman’s rank correlation coefficient.

Overall survival curves were estimated by the Kaplan–Meier method and compared by the two-sided log-rank test.

*p* values <0.05 were regarded as significant in all analyses. All analyses were performed using the statistical software Statistica 10 (Stat Soft Inc.).

## Results

### Immunohistochemistry for GrB+ cells

**Fig. 1:**
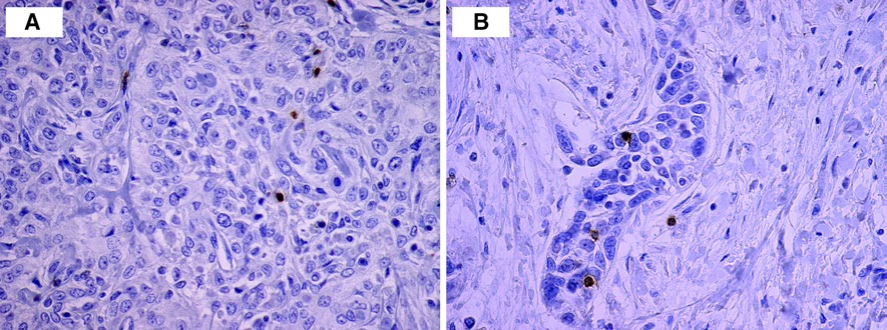
Microphotograph of immunohistochemical staining for CD56+ cells (**a**) and GrB+ cells (**b**) within the primary cancer nest

GrB+ cells were predominantly detected in the tumor stroma. They were less abundant within the cancer cell nests (Fig. 1a). For further study, the intraepithelial cell index was assessed.

The median number of IE GrB+ cells was 3 (range 0.00–13.33) per single high-power field (HPF). Patients in the advanced stage of the disease (35 metastatic cases) and in the locally limited stage (41 non-metastatic cases) had primary cancer nests that were similarly infiltrated by (IE) GrB+ cells (median 3.17 vs. 2.63, *p* = 0.07).

In 11 cases, no IE GrB+ cells were observed (14.47 %, GrB-negative tumors).

GrB-negative tumors exhibited a higher degree of morphological differentiation and were less frequently associated with lymph node metastases (Table 1).

**Table 1:** Comparison of clinicopathological features between patients having primary tumors negative and positive for GrB+ as well as CD56+ cells

Clinicopathological feature	IE GRB		*p*	IE CD56		*p*
	Negative (*n* = 11)	Positive (*n* = 61)		Negative (*n* = 10)	Positive (*n* = 65)	
Age /median/	62	70	0.134	74	69	0.554
depth of invasion /median/	6	7.03	0.204	6.63	7	0.291
G1/G2 + G3	8/3	16/45	**0.049**	4/6	22/43	0.731
G1/G2/G3	8/1/2	16/28/17	**0.009**	4/3/3	22/26/17	0.832
pT	10/1/0	55/5/1	0.909	8/2/0	60/4/1	0.306
meta+/meta−	2/9	32/29	**0.05**	5/5	29/36	1.0
FIGO stage I/II/III/IV	8/1/2/0	28/1/28/4	0.130	4/1/4/1	35/1/26/3	0.374
recurrence +/−	1/10	14/47	0.439	2/8	13/52	1.0
Overall survival			ns			ns

Bold values indicate statistical significance

The median number of GrB+ cells infiltrating lymph node metastases was 0.83 (range 0.00–8.33). A lack of intracarcinomatous GrB+ cells in nodal metastases was observed in 6 of 28 cases (21.43 %), whereas the primary tumors from these patients contained IE GrB+ cells. The lack of GrB+ cells within metastases does not influence overall survival (*p* = 0.512).

### Immunohistochemistry for CD56+ cells

CD56+ T cells were detected within cancer cell nests and sporadically in the mesenchymal stroma (Fig. 1b).

The median number of (IE) CD56+ cells was 2 (range 0.00–37) per single high-power field (HPF). Patients in the advanced stage of the disease (35 metastatic cases) had primary cancer nests that were more infiltrated by (IE) CD56+ cells than patients in the locally limited stage (41 non-metastatic cases) (median 2 vs. 1.67, *p* = 0.05).

A lack of IE CD56+ infiltrates was observed in 10 of 76 cases (13.16 %). This group did not correspond with the GrB-negative patients described above.

No differences were observed in all compared clinicopathological features between cases with and without (IE) CD56+ infiltrates (Table 1).

The median number of CD56+ T cells infiltrating lymph node metastases was 1.16 (range 0.00–10). A lack of CD56+ infiltrates within nodal metastases was observed in 8 of 35 cases (22.86 %). Only 2 patients had primary nests that were not infiltrated by IE CD56+ cells.

The lack of CD56+ cells in lymph node metastases had no impact on overall survival (*p* = 0.3012).

### Correlation of IE CD56+ and IE GrB+ cells with clinicopathological features

The number of IE CD56+ (per HPF) was correlated with the depth of invasion and recurrence, while the intensity of the IE GrB+ lymphocytes was correlated with age and tumor grade based on a 2-tier scale: G1 versus G2 + G3 (Table 2).

**Table 2:** Correlation of IE CD56+ and IE GrB+ infiltrates with clinicopathological features of vSCC patients

Clinicopathological feature	IE CD56+	*p*	IE GzB+	*p*
Age	*r* = −0.126	0.280	*r* = 0.333	**0.004**
pT	*r* = 0.001	0.992	*r* = 0.021	0.862
depth of invasion	*r* = 0.339	**0.003**	*r* = 0.196	0.102
G1/G2/G3	*r* = 0.023	0.849	*r* = 0.207	0.080
G1/G2 + G3	*r* = 0.093	0.432	*r* = 0.304	**0.009**
pN	*r* = 0.222	0.057	0.216	0.069
FIGO stage I/II/III/IV	*r* = 0.208	0.075	*r* = 0.226	0.056
recurrence +/−	*r* = −0.295	**0.011**	*r* = 0.109	0.362

Bold values indicate statistical significance

### Prognostic significance of IE GrB+ cells at the primary site

Patients were divided into GrB low- and high-intensity groups (low GrB+ and high GrB +, respectively) based on the median value of IE GrB+ cells.

With the exception of age (high GrB+ patients were older than low GrB+ patients), no differences were observed between groups in the pathological features analyzed here (Table 3).

**Table 3:** Differences in clinicopathological features between patients having primary tumors infiltrated with low and high numbers of intraepithelial GrB+ and CD56+ cells (median as a cutoff point)

Clinicopathological feature	Low (IE)GrB+	High (IE)GRB+	p	Low (IE)CD56+	High (IE)CD56+	p
Age /median/	65	71	**0.011**	69	69	0.996
depth of invasion /median/	7	7.4	0.355	6.25	7.75	0.01
G1/G2/G3	15/9/8	9/20/11	0.070	17/15/13	9/14/7	0.509
G1/G2 + G3	15/17	9/31	0.044	17/28	9/21	0.622
pT	30/1/1	35/5/0	0.202	41/3/1	27/3/0	0.632
meta+/meta−	12/20	22/18	0.161	17/28	17/13	0.155
FIGO stage I/II/III/IV	19/1/11/1	17/1/19/3	0.496	27/1/15/2	12/1/15/2	0.410
recurrence +/−	5/27	10/30	0.392	5/40	10/20	0.036

Bold values indicate statistical significance

**Fig. 2:**
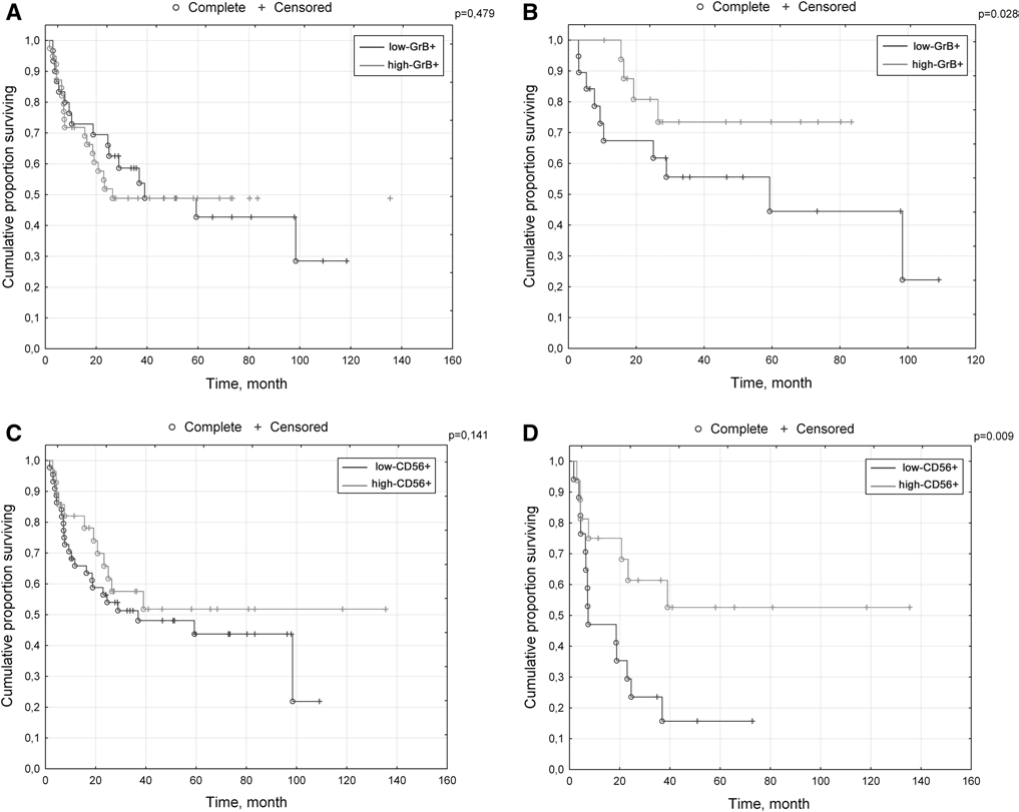
Kaplan–Meier survival curves for overall survival of patients. **a** low CD56+/high CD56+ infiltrates within cancer nests in the general population. **b** low CD56+/high CD56+ infiltrates within cancer nests in metastatic cases. **c** low GrB+/high GrB+ infiltrates within cancer nests in the general population. **d** low GrB+/high GrB+ infiltrates within cancer nests in non-metastatic cases

While no differences in overall survival (OS) were observed between high GrB+ and low GrB+ cases in the general cohort (Fig. 2a) (F Cox *p* = 0.479), more IE GrB+ cells than the median were correlated with longer OS among cases with local disease (F Cox *p* = 0.028) (Fig. 2b).

### Prognostic significance of IE CD56+ cells at the primary site

Patients were divided into CD56 low- and high-intensity groups (low CD56+ and high CD56+, respectively) based on the median value of IE CD56+ cells.

No differences were observed in all analyzed clinico-pathological features among different CD56 infiltration categories in our cohort (Table 3).

While no differences in overall survival were observed between these groups in the general cohort (F Cox *p* = 0.141) (Fig. 2c), intensities of IE CD56+ cells exceeding the median (high-CD56+) were correlated with longer OS among patients with metastatic dissemination (F Cox *p* = 0.009) (Fig. 2d).

## Discussion

The distribution of CD56+ cells and GrB expressing cells at the primary site differed in the vSCC cases analyzed here. CD56+ cells were mostly detected within the cancer nests, while the GrB+ cells were predominant in the tumor stroma. Only activated cytotoxic lymphocytes and NK/NKT cells are able to express granzyme B [12, 13]. The higher stromal distribution of GrB+ cells suggests that the vast majority of immune effectors are activated outside the cancer nests, chiefly through mechanisms unrelated to the recognition of vSCC antigens.

Moreover, the large discrepancy between IE GrB + infiltrates (median 3) and the cumulative number of intraepithelial immune effectors (median 2 for IE CD56+ and median 19 for IE CD8+ infiltrates) recently described by our group for this cohort [14] could suggest that the cytolytic power of the immune system has been lost outside rather than inside cancer nests.

This could explain the lack of prognostic significance of CD8+ lymphocytes in vSCC [15, 16] and suggests that the number of TILs present in the stroma probably provides a better indication of coexisting inflammatory processes than the immunogenicity of cancer cells.

In contrast, TILs within cancer nests are certainly connected with direct spontaneous immune reactions against cancer [20, 21], and therefore, only these intraepithelial infiltrates (IE) were analyzed further.

We found that the median numbers of IE GrB+ and IE CD56+ cells were comparable (3 and 2 per HPF within the primary cancer nest and 0.83 and 1.016 per HPF within lymph node metastases, respectively). This could suggest that these cells are the same cells, but considering the current results together with our previous data on IE CD8+ T lymphocytes in this cohort [14], we believe that only a small subset of IE CD56+ cells could be granzyme B positive.

This is supported by further results indicating that IE CD56+ and IE GrB+ infiltrates have separate correlations with clinico-pathological features in patients with vSCC.

The intensity of IE CD56+ cells was significantly correlated with depth of invasion and recurrence, while the intensity of IE GrB+ lymphocytes was correlated with age and tumor grade.

Patients in the advanced stage of the disease had primary cancer nests that were more infiltrated by IE CD56+ cells than cases in the locally limited stage. This was not observed for IE GrB+ infiltrates. These data could suggest that IE CD56+ infiltrates coincide with more aggressive tumors.

However, neither high IE CD56+ nor high IE GrB+ infiltrates had an impact on overall survival in vSCC patients. Further, to analyze the prognostic significance of these cells, we decided to subdivide the cohort into 2 groups: non-metastatic and metastatic. From the immunological point of view, patients with local and disseminated disease represent unsuccessful and successful immune escape, respectively [22]. Therefore, subdividing the general cohort into these two subtypes and conducting separate survival analyses seems to be justified.

High IE GrB+ infiltrates were correlated with longer OS in non-metastatic cases. It is unclear whether the immune surveillance performed by the cytotoxic function of the activated effectors of adaptive (CD8+ T cells) and innate (NK/NKT cells) immunity is efficient until tumors develop mechanisms of immune escape. Various proportions of the adaptive and innate effectors could be involved in granzyme B-dependent cytotoxicity because of the diverse expression of MHC antigens, costimulatory molecules and antigen-presenting cell (APC) statuses in different cancers, as well as the different number of regulatory cells within tumor tissue [23]. This could explain why the effectors of adaptive (CD8+) immunity did not reveal prognostic significance when analyzed separately [14].

Natural killer cells are considered to play a major role in the early stages of tumor development [11, 24, 25]. Previous reports showed that while innate immunity represents the first line of pathogen defense [26], most human tumor cells are resistant to perforin-mediated NK cell lysis, and NK cells are rarely found among TILs in advanced cancer cases [11].

Interestingly, the correlation between the intensity of IE CD56+ (NK/NKT) cells and survival was observed among metastatic vSCC patients, while the prognostic significance of granzyme B-dependent killing was not observed in this subgroup. Either these immune cells defending against metastases have already released GrB and are now GrB(−), or they represent a distinct subset of NK/NKT cells mediating tumor destruction by mechanisms other than GrB-mediated cytotoxicity. The second concept is consistent with more recent data suggesting that the primary biological role of NKs might not be the elimination of tumor targets, but rather the facilitation of dendritic-cell (DC)–T-cell interactions. As a consequence, this process may drive the immune responses against tumor-associated antigens (TAAs) [27]. This hypothesis could be supported by the fact that a diminished infiltration of CD4+ T cells is observed among advanced vSCC cases (authors’ unpublished data). CD4+ T-cell-deficient intratumoral infiltrates may impair the adaptive immune response, which is primarily regulated by DCs.

Recent data suggest that in addition to granzyme-mediated lysis, NK/NKT cells constitutively express several ligands of the TNF-family and can induce apoptosis in a broad variety of tumor-cell targets [28]. This mechanism might be of greater biological importance than secretory, granule-mediated killing, largely because of the expression of TNF-family ligands by tumor cells resulting in their sensitivity to apoptotic death [29].

Interestingly, patients with disseminated cancer were significantly older than patients without metastases in the cohort analyzed here [15]. It is known that immune anti-tumor responses can be influenced by the gradual deterioration of the immune system with age [10]. A significant expansion in the number of NK cells was observed upon aging [30], but some controversy remains concerning the efficacy of their cytotoxic function, which has been described as comparable to [31] or decreased [32, 33] compared with younger individuals.

## Conclusion

IE GrB+ infiltrates, which indicate the cytolytic power of combined adaptive and innate immune effectors, and IE CD56+ infiltrates, which indicate NK/NKT cells of unknown functional status, correlate with longer OS among non-metastatic and metastatic vSCC cases, respectively.

Both of these significant results suggest that immunological effects contribute to improved clinical outcomes in vSCC.
